# Increased Pulmonary Pneumococcal Clearance after Resolution of H9N2 Avian Influenza Virus Infection in Mice

**DOI:** 10.1128/IAI.00062-21

**Published:** 2021-05-17

**Authors:** Jingyun Li, Hongyan Wang, Pengjing Lian, Yu Bai, Zihui Zhang, Lihong Zhao, Tong Xu, Jian Qiao

**Affiliations:** a Department of Pathophysiology, College of Veterinary Medicine, China Agricultural University, Beijing, China; b Department of Veterinary Medicine, College of Animal Science, Hebei North University, Zhangjiakou, Hebei Province, China; University of California, Davis

**Keywords:** H9N2 virus, *Streptococcus pneumoniae*, secondary bacterial infections, pneumococcal clearance, mice

## Abstract

H9N2 avian influenza virus has been continuously circulating among poultry and can infect mammals, indicating that this virus is a potential pandemic strain. During influenza pandemics, secondary bacterial (particularly pneumococcal) pneumonia usually contributes to excessive mortality. In the present study, we observed the dynamic effect of H9N2 virus infection on host defense against secondary pneumococcal infection in mice. BALB/c mice were intranasally inoculated with 1.2 × 10^5^ PFU of H9N2 virus followed by 1 × 10^6^ CFU of Streptococcus pneumoniae at 7, 14, or 28 days post-H9N2 infection (dpi). The bacterial load, histopathology, body weight, and survival were assessed after pneumococcal infection. Our results showed that H9N2 virus infection had no significant impact on host resistance to secondary pneumococcal infection at 7 dpi. However, H9N2 virus infection increased pulmonary pneumococcal clearance and reduced pneumococcal pneumonia-induced morbidity after secondary pneumococcal infection at 14 or 28 dpi, as reflected by significantly decreased bacterial loads, markedly alleviated pulmonary histopathological changes, and significantly reduced weight loss in mice infected with H9N2 virus followed by S. pneumoniae compared with mice infected only with S. pneumoniae. Further, the significantly decreased bacterial loads were observed when mice were previously infected with a high dose (1.2 × 10^6^ PFU) of H9N2 virus. Also, similar to the results obtained in BALB/c mice, improvement in pulmonary pneumococcal clearance was observed in C57BL/6 mice. Overall, our results showed that pulmonary pneumococcal clearance is improved after resolution of H9N2 virus infection in mice.

## INTRODUCTION

H9N2 avian influenza virus has become widespread among poultry in many areas of Eurasia and Africa over the last 3 decades and was the dominant subtype isolated from chickens in China during 2016 to 2019 (1, 2). H9N2 virus has also been isolated from pigs, minks, and humans, demonstrating that this virus could cross species barriers to infect mammals (3–5). Several serological surveys have showed that 13.7% to 37.2% of people in China might have been infected with H9N2 virus (6, 7). Moreover, H9N2 virus contributes to the genesis of the novel H7N9, H10N8, and H5N6 viruses, which have been found to cause severe diseases and even fatalities in humans (8–12). The wide prevalence, enlarged range of mammalian hosts, and extensive genetic reassortment underscore the pandemic threat of H9N2 virus to human health (13).

Streptococcus pneumoniae, or pneumococcus, is a common inhabitant of the upper respiratory tract in approximately 20% to 90% of healthy children and 5% to 20% of healthy adults (14, 15). Defects in host defense, however, could alter the normal interactions between S. pneumoniae and host and enable S. pneumoniae to invade the lung, causing pneumonia. Pneumococcal pneumonia is still a major health problem worldwide despite interventions, including vaccines and antibiotics (16). Influenza virus infection is a well-recognized risk factor for pneumococcal pneumonia. Infections with influenza virus followed by bacteria, particularly S. pneumoniae, are associated with high morbidity and mortality, which is evident from previous influenza pandemics as well as from seasonal influenza epidemics (17–21). For example, the estimates from clinical and autopsy cases have shown that more than 95% and 50% of severe illnesses and deaths that occurred during the 1918 pandemic and 2009 pandemic, respectively, are due to secondary bacterial (especially pneumococcal) infections (17–20).

Mechanisms of increased susceptibility to secondary bacterial infections following influenza virus infection have been widely studied since the 1918 pandemic (22, 23). Data from animal models indicate that influenza virus infections facilitate bacterial transmission (24), colonization, and infection by impairing tracheal mucociliary clearance (25), damaging the airway epithelium to expose bacterial attachment sites (26, 27), and suppressing lung innate immunity (28). Defects in lung innate immune response, including the loss and dysfunction of alveolar macrophages (29, 30) and neutrophils (31, 32) and the dysregulation of cytokine productions (33), could play a key role in promoting secondary bacterial pneumonia.

However, secondary bacterial infections were mostly performed after a limited set of mouse-adapted laboratory influenza virus infections in previous studies. Secondary bacterial infections following other influenza virus infections have been less studied. Given the pandemic threat of H9N2 virus to human beings and the fact that secondary pneumococcal pneumonia accounts for excessive mortality during influenza pandemics, it is necessary to determine whether H9N2 virus infection predisposes hosts to secondary pneumococcal infection. Thus, the present study was designed to observe the effect of H9N2 virus infection on the host resistance to secondary pneumococcal infection at different time points post-H9N2 infection by utilizing mouse models. Understanding the interplay among H9N2 virus, host, and S. pneumoniae may provide better strategies for H9N2 pandemic preparedness. Here, our results showed that H9N2 virus infection did not increase the susceptibility of mice to secondary pneumococcal infection at 7 days post-H9N2 infection and improved pulmonary pneumococcal clearance when secondary pneumococcal infection was performed after resolution of H9N2 virus infection.

## RESULTS

### H9N2 virus infection caused obvious respiratory diseases in BALB/c mice.

**FIG 1:**
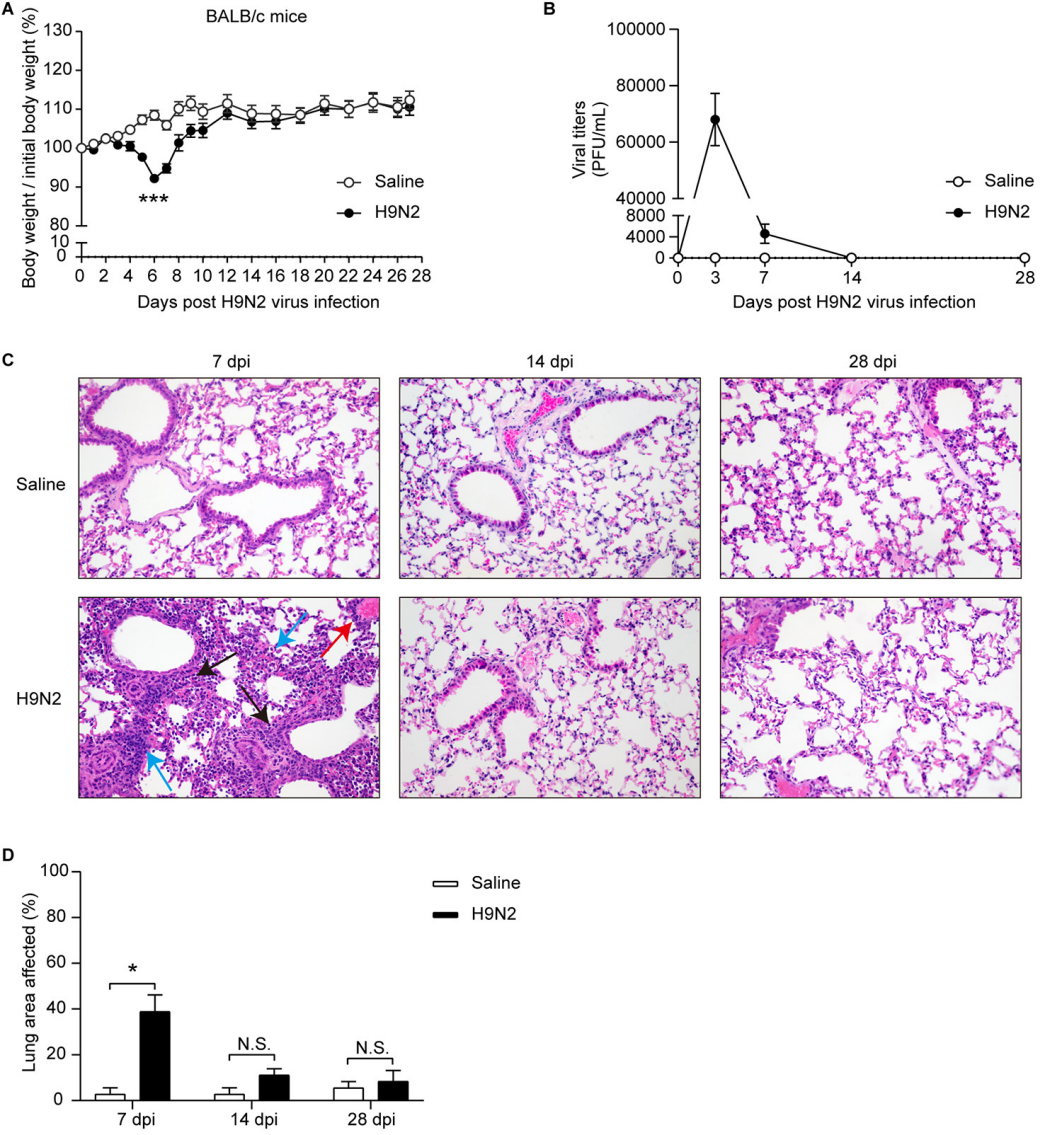
H9N2 virus infection caused obvious respiratory diseases in BALB/c mice. BALB/c mice were intranasally inoculated with 1.2 × 10^5^ PFU of H9N2 virus or with noninfectious allantoic fluid diluted in sterile saline as a control. (A) Body weight changes of mock-infected mice and H9N2-infected mice after H9N2 virus infection (*n* = 10/group). (B) Viral titers in the lungs of H9N2-infected mice at 3, 7, 14, and 28 dpi (*n* =2 or 3/group). (C) Representative H&E-stained lung sections (magnification, ×400) of mock-infected mice and H9N2-infected mice at 7, 14, and 28 dpi (*n* = 3/group). Extensive inflammatory cellular infiltration around bronchioles (black arrows), alveoli (blue arrows), and blood vessels (red arrows) was observed in the lungs of H9N2-infected mice at 7 dpi. (D) Percentage of lung areas affected in mock-infected mice and H9N2-infected mice, calculated from specified histopathological parameters, including peribronchial inflammation, intraalveolar inflammation and perivascular inflammation (*n* = 3/group). Data are means and SEM. Two-tailed unpaired Student’s *t* test was applied for two-group comparisons. *, *P* < 0.05; ***, *P* < 0.001; N.S., not significant.

After a nonlethal dose (1.2 × 10^5^ PFU) of H9N2 virus infection, BALB/c mice exhibited slight inactivity, chills, ruffled fur, and inappetence at 3 days post-H9N2 infection (dpi) and more severe clinical signs from 5 to 7 dpi. In addition, H9N2-infected mice showed gradual weight loss, reached peak weight loss at 6 dpi, and recovered gradually thereafter (Fig. 1A). The body weight was not significantly different between H9N2-infected mice and mock-infected mice at 10 dpi and afterward (Fig. 1A). The virus was detected in the lungs of H9N2-infected mice at 3 and 7 dpi but not at 14 or 28 dpi (Fig. 1B). Additionally, pronounced bronchiolitis and alveolitis, characterized by extensive inflammatory cellular infiltration around bronchioles, alveoli, and blood vessels, were seen in the lungs of H9N2-infected mice at 7 dpi (Fig. 1C). The overall architecture of the lungs of H9N2-infected mice was similar to those of mock-infected mice at 14 and 28 dpi (Fig. 1C). In line with these observations, the percentage of lung areas affected, determined by semiquantitative measurement of histopathological alterations, was significantly increased in H9N2-infected mice compared with mock-infected mice at 7 dpi but was not significantly different between the two groups at 14 and 28 dpi (Fig. 1D). Altogether, these data showed that H9N2 virus infection caused obvious respiratory diseases in BALB/c mice and that mice infected with H9N2 virus had recovered by 14 dpi.

### H9N2 virus infection had no significant impact on host resistance to secondary pneumococcal infection at 7 dpi in BALB/c mice.

**FIG 2:**
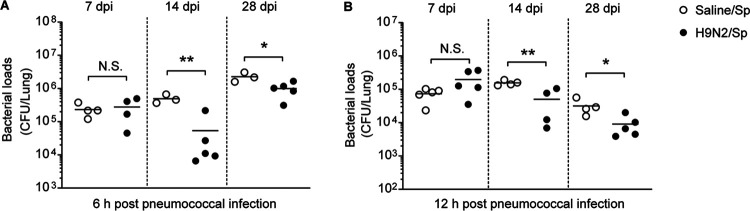
H9N2 virus infection improved pulmonary pneumococcal clearance in BALB/c mice when secondary pneumococcal infection was performed at 14 or 28 dpi. BALB/c mice were intranasally inoculated with 1.2 × 10^5^ PFU of H9N2 virus or with noninfectious allantoic fluid diluted in sterile saline as a control; at 7, 14, or 28 days after H9N2 virus infection, mice were intranasally inoculated with 1 × 10^6^ CFU of S. pneumoniae. Bacterial loads in the lungs of S. pneumoniae-infected mice and dually infected mice were determined at (A) 6 h and (B) 12 h after pneumococcal infection at 7, 14, or 28 dpi (*n* = 3 to 5/group). Data are means and SEM. Two-tailed unpaired Student’s *t* test was applied for two-group comparisons. *, *P* < 0.05; **, *P* < 0.01; N.S., not significant; Sp, Streptococcus pneumoniae.

It is well recognized that 7 days after influenza virus infection is a window of susceptibility to secondary bacterial infections in human and mouse models (34, 35). Therefore, we observed the effect of H9N2 virus infection on the host resistance to secondary pneumococcal infection at 7 dpi in BALB/c mice. As the ability of the lung to clear bacterial pathogens is an important part of the host defense against pulmonary bacterial infections, we first measured the pulmonary bacterial loads at 6 h and 12 h after infection with 1 × 10^6^ CFU of pneumococci at 7 dpi. No statistically significant differences in pulmonary bacterial loads were found between mice infected with H9N2 virus followed by S. pneumoniae (dually infected mice) and mice infected only with S. pneumoniae (S. pneumoniae-infected mice) (Fig. 2A and B, left).

**FIG 3:**
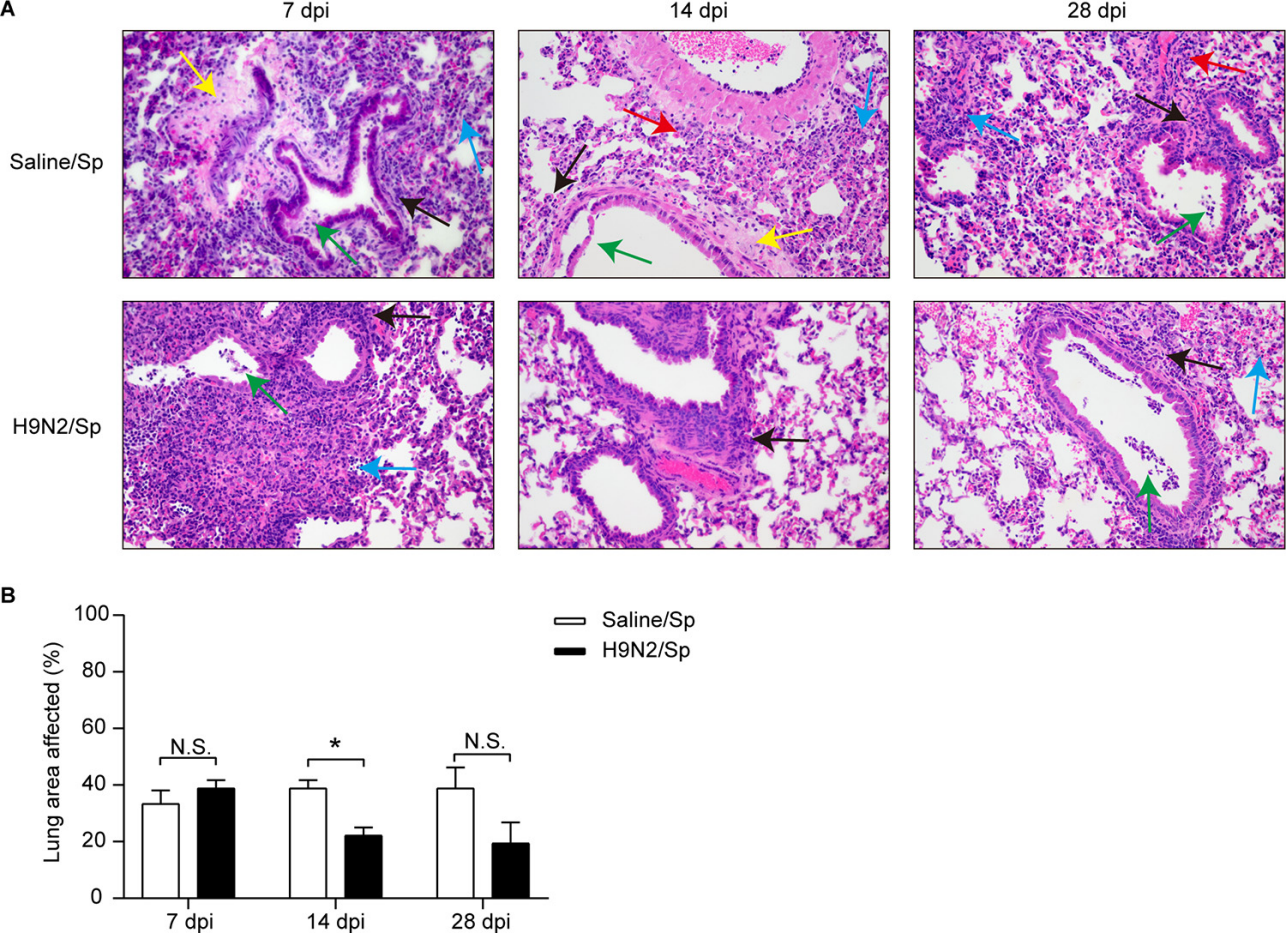
H9N2 virus infection alleviated pulmonary histopathological changes induced by pneumococcal infection at 14 dpi in BALB/c mice. (A) Representative H&E-stained lung sections (magnification, ×400) of S. pneumoniae-infected mice and dually infected mice at 6 h after pneumococcal infection at 7, 14, or 28 dpi (*n* = 3/group). Denuded epithelia (green arrows), intra-alveolar fibrin exudation (yellow arrows), and extensive inflammatory cell recruitment around bronchioles (black arrows), alveoli (blue arrows), and blood vessels (red arrows) were observed at 6 h after pneumococcal infection. (B) Percentage of lung areas affected in S. pneumoniae-infected mice and dually infected mice, calculated from specified histopathological parameters, including peribronchial inflammation, intra-alveolar inflammation, perivascular inflammation, bronchial epithelial shedding, and intra-alveolar fibrin exudation (*n* = 3/group). Data are means and SEM. Two-tailed unpaired Student’s *t* test was applied for two-group comparisons. *, *P* < 0.05; N.S., not significant. Sp, Streptococcus pneumoniae.

We then assessed lung histopathology at 6 h after pneumococcal infection at 7 dpi. Pronounced interstitial pneumonia, characterized by denuded epithelia, intra-alveolar fibrin exudation, and extensive inflammatory cell recruitment around bronchioles, alveoli, and blood vessels, was seen in the lungs of mice at 6 h after pneumococcal infection (Fig. 3A). The extents of pulmonary histopathological alterations were similar between dually infected mice and S. pneumoniae-infected mice (Fig. 3A and B).

**FIG 4:**
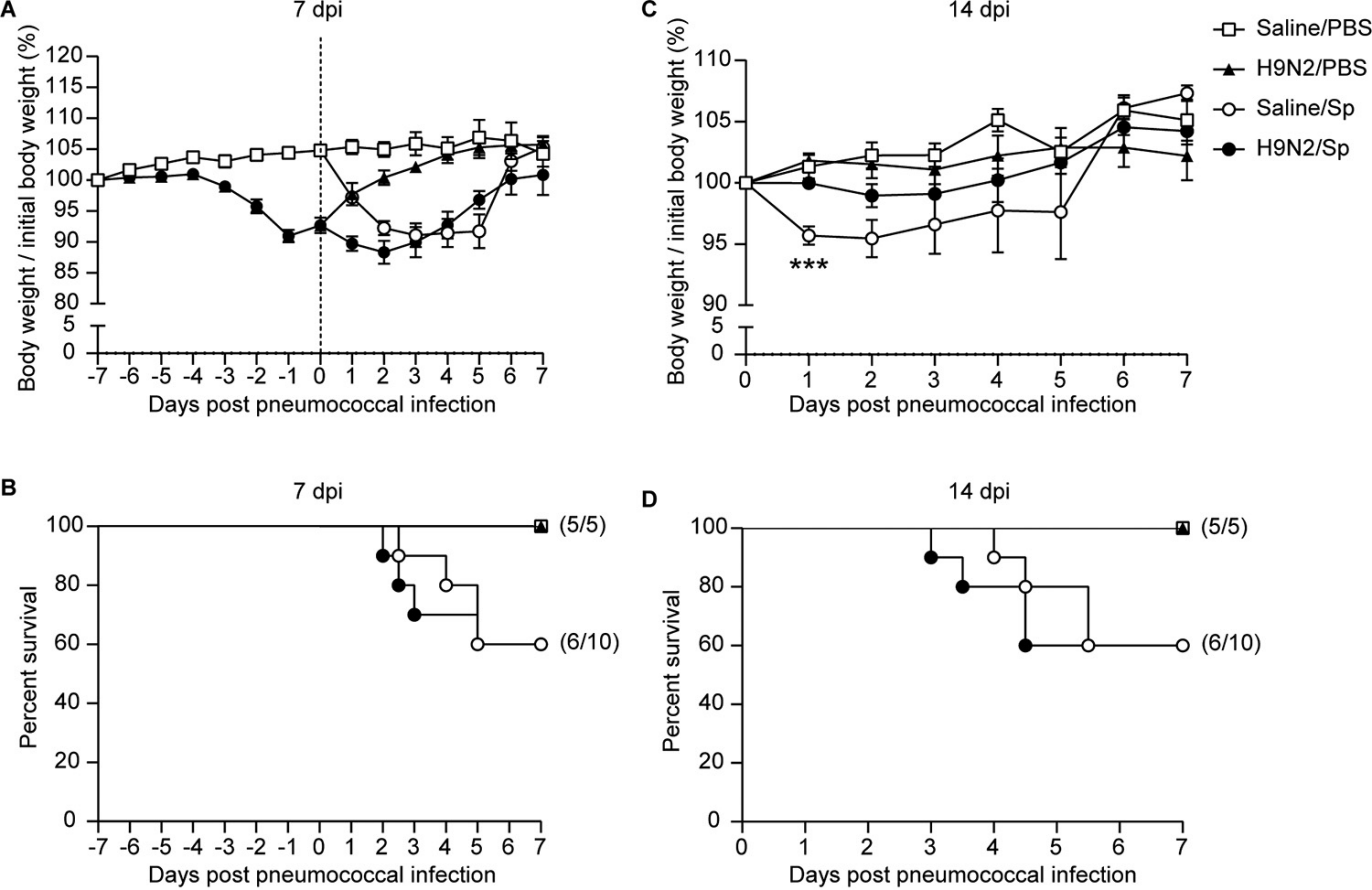
H9N2 virus infection did not change survival but reduced weight loss after secondary pneumococcal infection at 14 dpi in BALB/c mice. BALB/c mice were intranasally inoculated with 1.2 × 10^5^ PFU of H9N2 virus or with noninfectious allantoic fluid diluted in sterile saline as a control; 7 or 14 days after H9N2 virus infection, mice were intranasally inoculated with a 0.6 × median lethal dose (1 × 10^8^ CFU) of S. pneumoniae. Body weight changes (A and C) and survival (B and D) after pneumococcal infection at 7 dpi (A and B) or 14 dpi (C and D) are shown (mock-infected mice and H9N2-infected mice, *n* = 5/group; S. pneumoniae-infected mice and dually infected mice, *n* = 10/group). Data are means and SEM. Two-tailed unpaired Student’s *t* test was applied for body weight changes of two-group comparison, and a log-rank (Mantel-Cox) test was applied for survival comparison. ***, *P* < 0.001. PBS, phosphate-buffered saline; Sp, Streptococcus pneumoniae.

The body weight changes and survival were monitored after a 0.6 × median lethal dose (1 × 10^8^ CFU) of S. pneumoniae infection at 7 dpi. The body weight changes after pneumococcal infection were showed in Fig. 4A, and the degree of weight loss was not obviously different between dually infected mice and S. pneumoniae-infected mice (Table 1). The survival after pneumococcal infection was also not different between dually infected mice and S. pneumoniae-infected mice (Fig. 4B). Together, the above results showed that H9N2 virus infection had no significant impact on host resistance to secondary pneumococcal infection at 7 dpi in BALB/c mice.

**TABLE 1:** Weight loss after secondary pneumococcal infection at 7 days after H9N2 virus infection

Group	% weight loss on day after pneumococcal infection^*a*^						
	1	2	3	4	5	6	7
S. pneumoniae-infected mice	8.18	12.76	14.81	13.69	15.20	3.28	−1.04
Dually infected mice	8.03	12.08	12.20	11.41	8.48	5.47	4.89

a Calculated as mean percent of body weight in mock-infected mice − mean percent of body weight in S. pneumoniae-infected mice or mean percent of body weight in H9N2-infected mice − mean percent of body weight in dually infected mice.

### Preceding H9N2 virus infection increased pulmonary pneumococcal clearance and reduced pneumococcal pneumonia-induced morbidity in BALB/c mice after recovery from influenza.

We also observed the effect of H9N2 virus infection on the host resistance to secondary pneumococcal infection at 14 or 28 dpi, when mice infected with H9N2 virus had recovered from influenza. Bacterial loads at 6 h and 12 h after pneumococcal infection at 14 dpi were both significantly decreased in the lungs of dually infected mice compared with those in S. pneumoniae-infected mice (Fig. 2A and B, middle). Similarly, bacterial loads at 6 h and 12 h after pneumococcal infection at 28 dpi were both significantly decreased in the lungs of dually infected mice compared with S. pneumoniae-infected mice (Fig. 2A and B, right).

Markedly improved lung lesions with reduced inflammatory infiltrates were observed in the lungs of dually infected mice compared with S. pneumoniae-infected mice at 6 h after pneumococcal infection at 14 dpi (Fig. 3A). In line with these observations, the percentage of lung areas affected was significantly decreased in dually infected mice compared with S. pneumoniae-infected mice at 6 h after pneumococcal infection at 14 dpi (Fig. 3B). Similar results were also observed at 6 h after pneumococcal infection at 28 dpi, though a statistical significance decrease in the percentage of lung areas affected in dually infected mice compared with S. pneumoniae-infected mice was not obtained (Fig. 3A and B).

The body weight changes and survival were also monitored after a 0.6 × median lethal dose (1 × 10^8^ CFU) of S. pneumoniae infection at 14 dpi. Weight loss was significantly reduced 1 day after pneumococcal infection in dually infected mice compared with S. pneumoniae-infected mice (Fig. 4C). The survival after pneumococcal infection was not different between dually infected mice and S. pneumoniae-infected mice (Fig. 4D). Collectively, the above results demonstrated that prior H9N2 virus infection increased pulmonary pneumococcal clearance and reduced pneumococcal pneumonia-induced morbidity in BALB/c mice after recovery from influenza.

### H9N2 virus infection modulated pulmonary chemokine and cytokine responses to subsequent pneumococcal infection.

To determine the pulmonary chemokine and cytokine responses to secondary pneumococcal infection following H9N2 virus infection, we measured the levels of chemokines (keratinocyte chemoattractant [KC] and mouse macrophage inflammatory protein-2 [MIP-2]), the anti-inflammatory cytokine interleukin-10 (IL-10), and proinflammatory cytokines (IL-6, tumor necrosis factor alpha [TNF-α], and IL-1β) at 6 h after pneumococcal infection at 7, 14, or 28 dpi.

**FIG 5:**
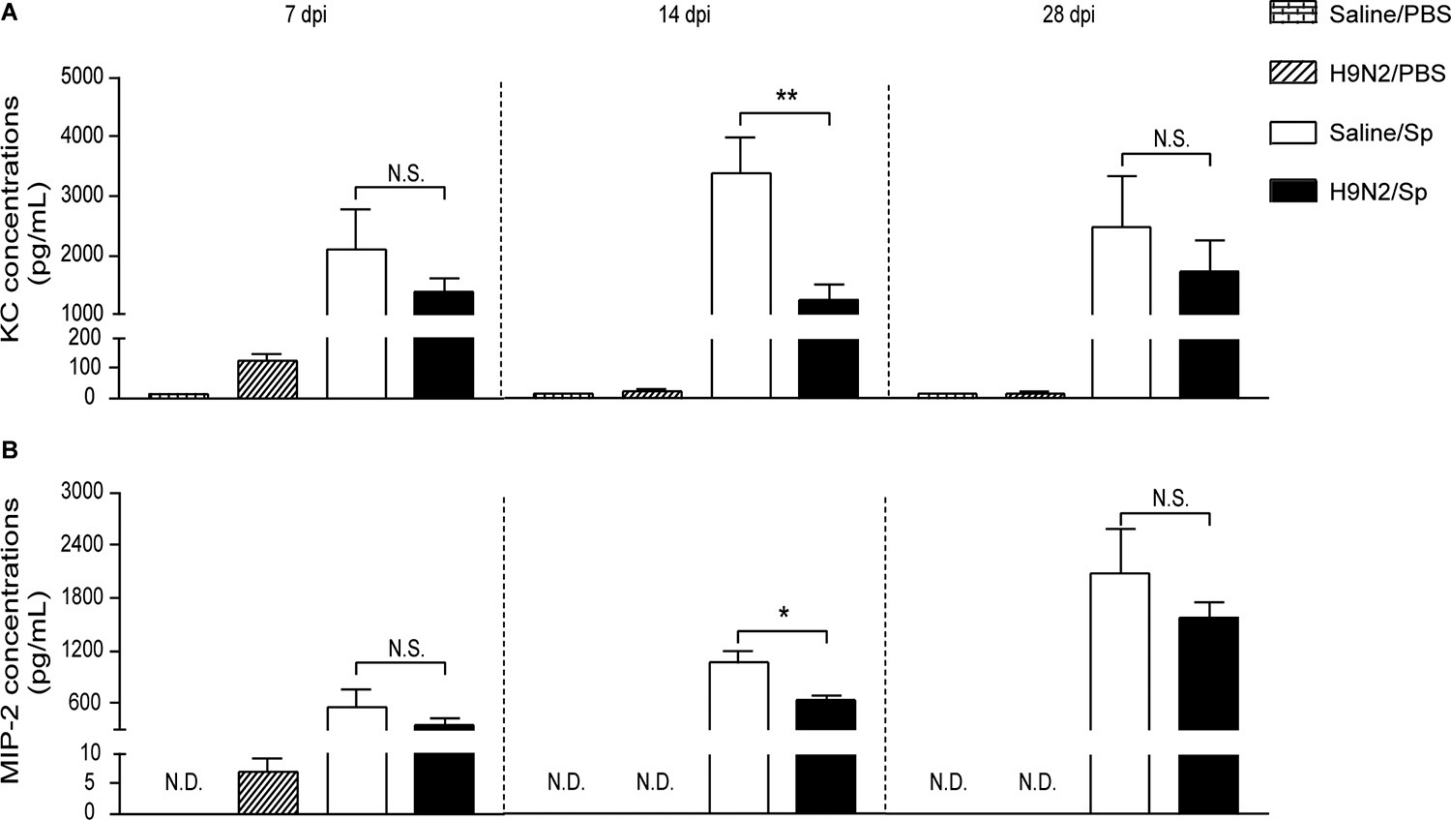
H9N2 virus infection reduced productions of KC and MIP-2 after pneumococcal infection at 14 dpi. Concentrations of (A) KC and (B) MIP-2 in the lungs of mock-infected mice, H9N2-infected mice, S. pneumoniae-infected mice, and dually infected mice at 6 h after pneumococcal infection at 7 dpi (left), 14 dpi (middle), or 28 dpi (right) (*n* = 3 to 5/group). Data are means and SEM. Two-tailed unpaired Student’s *t* test was applied for two-group comparisons. *, *P* < 0.05; **, *P* < 0.01; N.S., not significant. N.D., not detectable; KC, keratinocyte chemoattractant; MIP-2, mouse macrophage inflammatory protein-2; PBS, phosphate-buffered saline; Sp, Streptococcus pneumoniae.

**FIG 6:**
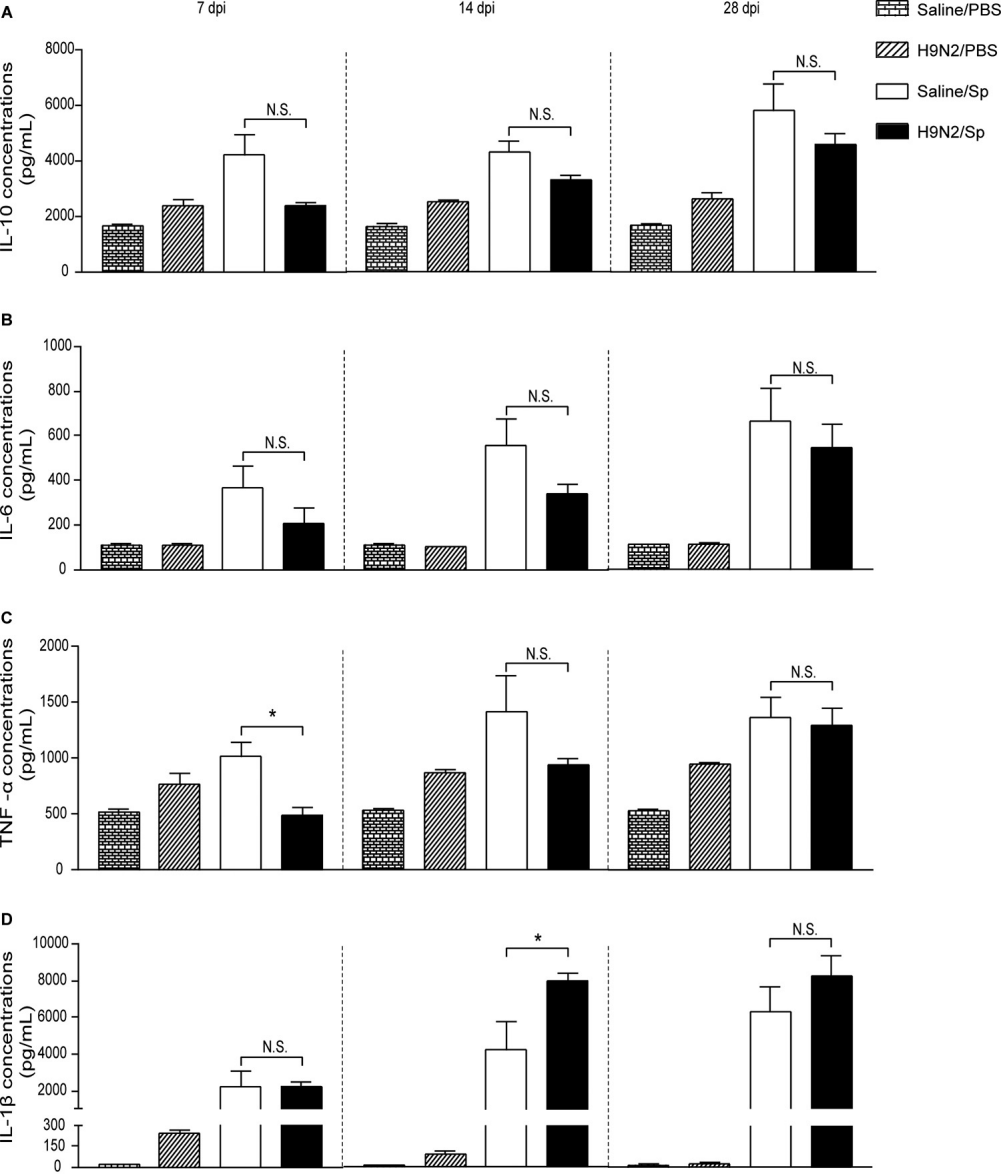
H9N2 virus infection reduced TNF-α production after pneumococcal infection at 7 dpi and promoted IL-1β production after pneumococcal infection at 14 dpi. Concentrations of (A) IL-10, (B) IL-6, (C) TNF-α, and (D) IL-1β in the lungs of mock-infected mice, H9N2-infected mice, S. pneumoniae-infected mice, and dually infected mice at 6 h after pneumococcal infection at 7 dpi (left), 14 dpi (middle), or 28 dpi (right) (*n* = 3 to 5/group). Data are means and SEM. Two-tailed unpaired Student’s *t* test was applied for two-group comparisons. *, *P* < 0.05; N.S., not significant. IL-10, interleukin-10; IL-6, interleukin-6; TNF-α, tumor necrosis factor alpha; IL-1β, interleukin-1β; PBS, phosphate-buffered saline; Sp, Streptococcus pneumoniae.

After pneumococcal infection at 7 dpi, the levels of KC and MIP-2 were similar in the lungs of dually infected mice and S. pneumoniae-infected mice (Fig. 5A and B, left). The levels of IL-10, IL-6, and IL-1β were also similar in the lungs of dually infected mice and S. pneumoniae-infected mice (Fig. 6A, B, and D, left). However, the TNF-α levels were significantly decreased in the lungs of dually infected mice compared with S. pneumoniae-infected mice (Fig. 6C, left). These data showed that H9N2 virus infection reduced TNF-α production after pneumococcal infection at 7 dpi.

After pneumococcal infection at 14 dpi, the levels of KC and MIP-2 were both significantly decreased in the lungs of dually infected mice compared with S. pneumoniae-infected mice (Fig. 5A and B, middle). The levels of IL-10, IL-6, and TNF-α were similar in the lungs of dually infected mice and S. pneumoniae-infected mice (Fig. 6A to C, middle). However, the IL-1β levels were significantly increased in the lungs of dually infected mice compared with S. pneumoniae-infected mice (Fig. 6D, middle). After pneumococcal infection at 28 dpi, no statistically significant differences were found in the levels of these chemokines and cytokines between dually infected mice and S. pneumoniae-infected mice (Fig. 5A and B and 6A to D, right). Taken together, these results suggested that H9N2 virus infection reduced production of KC and MIP-2 but promoted IL-1β production after pneumococcal infection at 14 dpi.

### A high dose of H9N2 virus infection also promoted pulmonary pneumococcal clearance.

**FIG 7:**
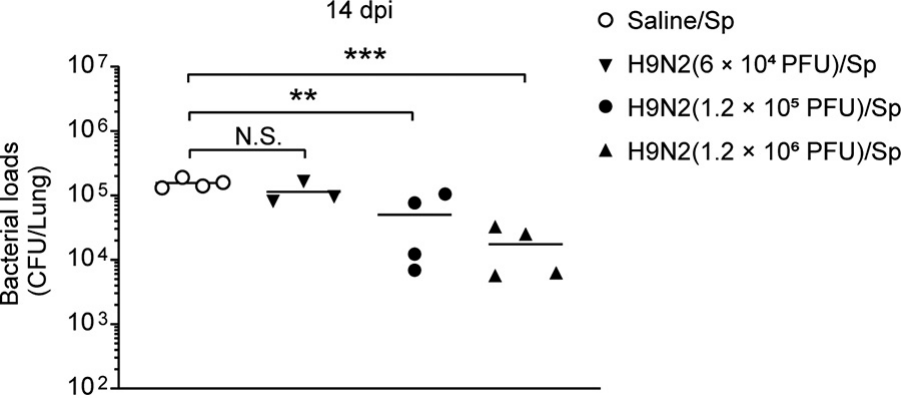
A high dose of H9N2 virus infection also promoted pulmonary pneumococcal clearance. BALB/c mice were intranasally inoculated with 6 × 10^4^ PFU (low dose), 1.2 × 10^5^ PFU, or 1.2 × 10^6^ PFU (high dose) of H9N2 virus or with noninfectious allantoic fluid diluted in sterile saline as a control; 14 days after H9N2 virus infection, all mice were intranasally inoculated with 1 × 10^6^ CFU of S. pneumoniae. Pulmonary bacterial loads at 12 h after pneumococcal infection were measured (*n* = 3 or 4/group). Data are means and SEM. Ordinary one-way ANOVA followed by Tukey’s multiple-comparison test was applied for four-group comparisons. **, *P* < 0.01; ***, *P* < 0.001; N.S., not significant. Sp, Streptococcus pneumoniae.

Different doses of H9N2 virus infection might have different effects on the ability of the lung to clear S. pneumoniae. Therefore, we measured the bacterial loads at 12 h after pneumococcal infection following a low dose (6 × 10^4^ PFU) or a high dose (1.2 × 10^6^ PFU) of H9N2 virus. The significantly decreased bacterial loads at 12 h after pneumococcal infection at 14 dpi were also observed when mice were previously infected with the higher dose but not the lower dose, of H9N2 virus (Fig. 7). Thus, these data showed that the higher dose of H9N2 virus also promoted pulmonary pneumococcal clearance.

### H9N2 virus infection increased pulmonary pneumococcal clearance in C57BL/6 mice after recovery from influenza.

**FIG 8:**
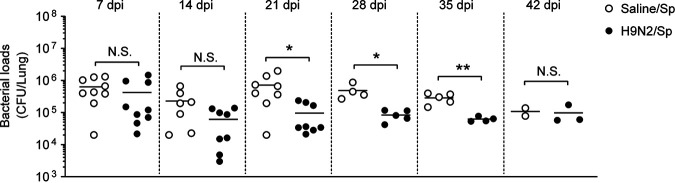
H9N2 virus infection increased pulmonary pneumococcal clearance in C57BL/6 mice when secondary pneumococcal infection was performed at 21, 28, or 35 dpi. C57BL/6 mice were intranasally inoculated with 1.2 × 10^5^ PFU of H9N2 virus or with noninfectious allantoic fluid diluted in sterile saline as a control; 7, 14, 21, 28, 35, 42 days after H9N2 virus infection, mice were intranasally inoculated with 1 × 10^6^ CFU of S. pneumoniae. Pulmonary bacterial loads at 6 h after pneumococcal infection were measured (*n* = 2 to 9/group). Data are means and SEM. Two-tailed unpaired Student’s *t* test were applied for two-group comparisons: *, *P* < 0.05; **, *P* < 0.01; N.S., not significant. Sp, Streptococcus pneumoniae.

Previous studies have shown that BALB/c mice and C57BL/6 mice differ in their susceptibility to pneumococcal infection (36, 37). To determine whether the effect of H9N2 virus infection on pulmonary pneumococcal clearance was mouse strain specific, we measured the bacterial loads at 6 h after pneumococcal infection following H9N2 virus infection in C57BL/6 mice. Bacterial loads were similar in the lungs of dually infected mice and S. pneumoniae-infected mice after pneumococcal infection at 7 dpi (Fig. 8). In addition, bacterial loads showed a tendency to decrease in the lungs of dually infected mice compared with S. pneumoniae-infected mice after pneumococcal infection at 14 dpi, though this difference did not achieve statistical significance (Fig. 8). Further, bacterial loads were significantly decreased in the lungs of dually infected mice compared with S. pneumoniae-infected mice after pneumococcal infection at 21 dpi (Fig. 8). Similar results were obtained after pneumococcal infection at 28 and 35 dpi but not at 42 dpi (Fig. 8). Therefore, these results showed that H9N2 virus infection also increased pulmonary pneumococcal clearance in C57BL/6 mice after recovery from influenza.

## DISCUSSION

H9N2 virus has been considered a potential pandemic strain due to its wide prevalence, extended range of mammalian hosts, and extensive genetic reassortment (13). Our results showed that a nonlethal dose (1.2 × 10^5^ PFU) of H9N2 virus infection caused obvious signs of illness and significantly decreased body weight from 3 to 7 dpi in BALB/c mice. The virus was detected in the lungs of H9N2-infected mice at 3 and 7 dpi, and extensive inflammatory cellular infiltration was observed in the lungs of H9N2-infected mice at 7 dpi. Further, there were no significant differences between the H9N2-infected mice and mock-infected mice concerning the clinical signs and body weight at 14 dpi. The virus had been completely eliminated from the lungs of H9N2-infected mice by 14 dpi, and lung histopathology in H9N2-infected mice was similar to that in mock-infected mice at 14 dpi. These results showed that H9N2 virus infection caused obvious respiratory diseases in BALB/c mice and mice infected with H9N2 virus had recovered by 14 dpi, which was consistent with our previous and other published research findings (38–40).

Although influenza virus alone can have a substantial impact on global health, secondary bacterial infections postinfluenza are associated with increased morbidity and mortality during both epidemic and pandemic influenza outbreaks (23). Secondary bacterial pneumonia, particularly due to S. pneumoniae, accounted for more than 95% and 50% of severe illnesses and deaths that occurred during the 1918 pandemic and 2009 pandemic, respectively (17–20). Therefore, it is important to understand the interactions among influenza virus, host, and bacteria. Most previous studies utilizing mouse models showed that influenza virus infections could increase host susceptibility to secondary bacterial infections around 7 days postinfluenza by decreasing lung defense (29, 30, 41). Results from the study by Chockalingam et al. showed that H9N2 virus (A/Duck/Hong Kong/702/1979) infection increased susceptibility of BALB/c mice to secondary pneumococcal infection at 7 days postinfluenza in terms of pulmonary bacterial loads, degree of weight loss, and survival (42). However, our results revealed that H9N2 virus (A/Chicken/Hebei/4/2008) infection did not increase the susceptibility of BALB/c mice to secondary pneumococcal infection at 7 dpi with respect to bacterial loads, lung histopathology, degree of weight loss, and survival. The disparate results between the study by Chockalingam et al. (42) and the present study might be due to the different strains of H9N2 virus being used. It has been proposed that several virulence factors of influenza virus have viral-strain-specific effects on the host that contribute to secondary bacterial pneumonia (43). Influenza viruses with functional PB1-F2 proteins or decreased glycosylation of surface proteins are thought to effectively facilitate subsequent bacterial infections (23). Additionally, high-activity neuraminidase of influenza viruses could cleave sialic acid receptors more effectively to expose bacterial attachment receptors and enable bacteria to cause disease (41, 44). Results from the study of Peltola et al. showed that the neuraminidase activity of H9N2 virus from chicken was very low (44), which may help explain the fact that H9N2 virus (isolated from chicken) infection did not promote secondary pneumococcal infection at 7 dpi in our study.

Increased susceptibility of mice to secondary pneumococcal infection was also observed when mice were challenged with S. pneumoniae after recovery from influenza in numerous previous studies. For example, H1N1 and H3N2 virus infection was shown to cause significantly increased pulmonary bacterial loads and mortality after pneumococcal infection at 14 days postinfluenza in mice (45–48). In addition, a study performed by Didierlaurent et al. demonstrated that H3N2 virus infection could still lead to significantly increased pulmonary bacterial loads and mortality after pneumococcal infection at 42 days postinfluenza in mice (46). In contrast, our results showed that H9N2 virus infection caused significantly decreased bacterial loads after pneumococcal infection at 14 or 28 dpi, suggesting that prior H9N2 virus infection increased pulmonary pneumococcal clearance in mice after recovery from influenza. Consistent with decreased bacterial loads, markedly alleviated pulmonary histopathological changes were also observed after pneumococcal infection at 14 dpi. In addition, H9N2 virus infection led to significantly reduced weight loss but did not change the mortality after pneumococcal infection at 14 dpi. These results implied that the effect of H9N2 virus infection on increasing the host resistance to pneumococcal infection at 14 dpi is limited; i.e., it could reduce secondary pneumococcal pneumonia-induced morbidity but was not sufficient to decrease secondary pneumococcal pneumonia-induced mortality in BALB/c mice.

We also determined whether the dose of H9N2 virus impacts the pulmonary pneumococcal clearance. Our result showed that the significantly decreased bacterial loads after pneumococcal infection were also observed when mice were infected 14 days previously with a high dose but not a low dose of H9N2 virus. Thus, the higher dose of H9N2 virus also promoted pulmonary pneumococcal clearance. Moreover, the beneficial effect of H9N2 virus infection on pulmonary pneumococcal clearance was not dependent on active viral replication, as the virus had been completely eliminated from the lungs of H9N2-infected mice by 14 dpi.

It has been reported that C57BL/6 mice are more susceptible to pneumococcal infection than BALB/c mice (36, 37). In the present study, our results showed that, similar to the results obtained in BALB/c mice, H9N2 virus infection led to significantly decreased bacterial loads after pneumococcal infection at 21, 28, and 35 dpi in C57BL/6 mice, suggesting that the beneficial effect of H9N2 virus infection on pulmonary pneumococcal clearance was not mouse strain specific.

Recently, similar to our results, Aegerter et al. found that H3N2 virus preceding 28-day infection also increased host resistance to secondary pneumococcal infection in terms of bacterial loads and mortality in C57BL/6 mice, and this prolonged antibacterial protection was attributed to a population of monocyte-derived alveolar macrophages that produce increased IL-6 (49). During pneumococcal infection, neutrophils also play a key role in eliminating S. pneumoniae (50). The exact roles of macrophages and neutrophils underlying the protection against secondary pneumococcal infection conferred by H9N2 virus infection would need to be investigated in further studies.

It is generally assumed that local production of chemokines and cytokines, as an important part of the innate immune response against bacterial infections, might play a role in the clearance of bacterial pathogens. However, a study done by Dallaire et al. showed that the levels of KC, MIP-2, IL-6, and IL-1β in lungs of mice with pneumococcal infection were positively correlated with bacterial load (51). It was also shown that the production of KC, IL-10, IL-6, TNF-α, and IL-1β was significantly enhanced when bacterial loads were also significantly increased after S. pneumoniae infection at 7 days after H9N2 virus (A/Duck/Hong Kong/702/1979) infection (42). Our results showed that the production of KC, MIP-2, IL-10, IL-6, and IL-1β did not significantly change when bacterial loads were similar in the lungs of dually infected mice and S. pneumoniae-infected mice after pneumococcal infection at 7 dpi. These results might be explained by the fact that production of these mediators is dependent, at least in part, on direct stimulation by S. pneumoniae. However, TNF-α production was significantly reduced in the lungs of dually infected mice compared with S. pneumoniae-infected mice when bacterial loads were similar between the two groups after pneumococcal infection at 7 dpi in our study. Results from the study of Kirby et al. showed that TNF-α was produced mainly by alveolar macrophages during pneumococcal pneumonia but was not essential for pneumococcal clearance (52). Thus, the significantly decreased TNF-α production in the present study might be explained by the fact that H9N2 virus preceding 7-day infection limited the ability of alveolar macrophages to produce TNF-α without impacting pneumococcal clearance.

When secondary pneumococcal infections were performed at 14 dpi, the production of KC and MIP-2 was significantly reduced when bacterial loads were significantly decreased in the lungs of dually infected mice compared with S. pneumoniae-infected mice in our study. Conversely, KC production was significantly enhanced when bacterial loads were significantly increased after pneumococcal infection at 14 days after H1N1 infection or at 14 days after H3N2 infection (47, 48). These results also suggested that production of KC and MIP-2 after secondary pneumococcal infection at 14 days after influenza was associated with the direct stimulation by S. pneumoniae. In addition, the production of IL-10, IL-6, and TNF-α did not significantly change, notably in contrast to IL-1β, which was significantly enhanced after pneumococcal infection at 14 dpi in our study. Recently, enhanced IL-1β production was shown to be associated with induction of trained immunity (53–55). The concept of trained immunity is proposed to describe the fact that long-term activation of innate immune responses by certain pathogens or live vaccines could confer nonspecific protection against subsequent infections by dissimilar pathogens (56). Taking this into account, the beneficial effect of H9N2 virus infection on pulmonary pneumococcal clearance might partially be due to the induction of trained immunity associated with the enhanced IL-1β production. Since bacterial loads were significantly decreased but IL-1β production did not significantly change after pneumococcal infection at 28 dpi, other cellular and soluble mediators might be involved in improving bacterial clearance during secondary pneumococcal infection following resolution of H9N2 virus infection. Further investigation would be required to clarify the induction of trained immunity by H9N2 virus infection.

In conclusion, our study shows that H9N2 virus infection did not enhance the susceptibility of mice to secondary pneumococcal infection at 7 days after H9N2 virus infection, and increased pulmonary pneumococcal clearance was seen upon secondary pneumococcal infection after resolution of H9N2 virus infection. The interactions among influenza virus, host, and S. pneumoniae are complex, and the effects of other influenza virus infections on susceptibility to secondary pneumococcal infection need to be investigated in further studies.

## MATERIALS AND METHODS

### Mouse strains.

Specific-pathogen-free (SPF) male BALB/c mice and C57BL/6 mice, all of which were between 6 and 8 weeks of age and weighed 18 to 20 g, were purchased from Beijing Vital River Laboratory Animal Technology Company Limited (China). All mouse experiments were approved by the Laboratory Animal Welfare and Animal Experimental Ethical Committee of China Agricultural University (no. AW12210202-2). All mice were acclimatized for 7 days before experimental treatments and had free access to food and water during the experiments.

### Viral and bacterial strains.

The H9N2 virus [A/Chicken/Hebei/4/2008(H9N2)] used in this study is one of the representative H9N2 isolates in northern China (57). The complete genome sequences of the virus are available in GenBank under accession numbers FJ499463 to FJ499470. Its pathogenicity in mice was assessed in detail in our previous study, and the results showed that this H9N2 virus infection caused severe lung injury with a high mortality without prior adaptation (38). The virus was propagated in the allantoic cavities of 10-day-old embryonated SPF chicken eggs at 37°C for 72 h, and then the allantoic fluid was centrifuged and stored at −80°C for use in all of the experiments described herein. For H9N2 viral inoculation, the frozen virus liquid was thawed and diluted in sterile saline. Actual H9N2 virus concentration was determined by plaque assay as described below. Results were expressed as PFU per milliliter.

S. pneumoniae (NCTC7466, serotype 2) was grown in Todd-Hewitt broth supplemented with 0.5% yeast extract broth at 37°C. When cultured at mid-log phase (optical density at 600 nm [OD_600_] = 0.3 to 0.4), the pneumococcal culture maintained in broth plus 20% glycerol was stored at −80°C for use in all of the experiments described herein (58). For S. pneumoniae inoculation, the frozen stock was thawed and cultured in broth at 37°C until mid-logarithmic phase. Then the culture was centrifuged, washed twice in sterile phosphate-buffered saline (PBS), and subsequently pelleted before dilution to the desired concentration. Actual pneumococcal concentration was determined by plating 0.1 ml of 10-fold serial dilutions on blood agar plates, and colonies were counted after incubation for 24 h at 37°C. Results were expressed as CFU per milliliter.

### Viral and bacterial inoculation.

Mice were lightly anesthetized by inhalation of isoflurane and then received a volume of 50 μl of viral or bacterial suspension at the tip of the nose, which was involuntarily inhaled. To facilitate the migration of the inoculum to the lung, mice were held in an upright position for 1 min. As a control, mice were mock infected with 50 μl of noninfectious allantoic fluid or sterile PBS in an identical manner.

### Experimental protocol.

**FIG 9:**
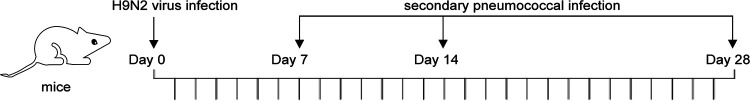
Schematic diagram of H9N2 virus infection and secondary pneumococcal infection. Secondary pneumococcal infections were mainly performed at 7, 14, or 28 days after H9N2 virus infection in mice.

The present study was designed to observe the effect of H9N2 virus infection on host resistance to secondary pneumococcal infection at different time points after H9N2 virus infection (Fig. 9) and was performed in three parts, as follows.

In the first part, two experiments were carried out. The first experiment was to observe the effect of H9N2 virus infection on the bacterial loads, lung histopathology, and cytokine levels after pneumococcal infection. BALB/c mice were intranasally inoculated with 1.2 × 10^5^ PFU of H9N2 virus or with noninfectious allantoic fluid as a control. Seven, 14, or 28 days after H9N2 virus infection, mice were intranasally inoculated with 1 × 10^6^ CFU of S. pneumoniae or with sterile PBS as a control (experimental mouse groups are shown in Table 2). The dose of 1.2 × 10^5^ PFU of H9N2 virus was chosen, as a pilot experiment had indicated that mice infected with 1.2 × 10^5^ PFU of H9N2 viruses would be ill but not dead and could be used to conduct secondary pneumococcal inoculation after H9N2 virus infection. After H9N2 virus infection, clinical signs and body weight, as measures of morbidity, were monitored daily; viral titers were measured at 3, 7, 14, and 28 dpi, and lung histopathology was assessed at 7, 14, and 28 dpi in mock-infected mice and H9N2-infected mice. After pneumococcal infection at 7, 14, or 28 dpi, the four groups of mice were sacrificed at 6 h or 12 h, and the whole lung tissues were harvested to analyze the lung histopathology, bacterial loads, and cytokine levels as described below.

**TABLE 2:** Experimental mouse groups after secondary pneumococcal infection

Group	Primary inoculation	Secondary inoculation
Mock infected	Noninfectious allantoic fluid	Sterile PBS
H9N2 infected	H9N2 virus	Sterile PBS
S. pneumoniae infected	Noninfectious allantoic fluid	S. pneumoniae
Dually infected	H9N2 virus	S. pneumoniae

The second experiment was to observe the effect of H9N2 virus infection on the body weight changes and survival after pneumococcal infection. BALB/c mice were also randomized into four groups as described in Table 2. H9N2 virus infection was performed as described for the first experiment, and secondary pneumococcal infection was performed by intranasally inoculating mice with a 0.6 × median lethal dose (1 × 10^8^ CFU) of S. pneumoniae at 7 or 14 dpi. Body weight was monitored daily, and survival was recorded every 12 h until 7 days after pneumococcal infection, when no more deaths were observed. Mice were euthanized when they had lost over 25% of their initial weight and appeared moribund (based on inability to move freely and access food and water) and were considered to have died on that day (42).

In the second part, we determined whether the dose of H9N2 virus impacts the pulmonary pneumococcal clearance. BALB/c mice were intranasally inoculated with 6 × 10^4^ PFU (low dose), 1.2 × 10^5^ PFU, or 1.2 × 10^6^ PFU (high dose) of H9N2 virus, and then all were intranasally inoculated with 1 × 10^6^ CFU of S. pneumoniae at 14 dpi. The mice were sacrificed at 12 h after pneumococcal infection and the whole lung tissues were harvested to analyze the bacterial load as described below.

In the third part, we determine whether the effect of H9N2 virus infection on pulmonary pneumococcal clearance was mouse strain specific, as previous studies have shown that BALB/c mice and C57BL/6 mice differ in their susceptibility to pneumococcal infection (36, 37). C57BL/6 mice were intranasally inoculated with 1.2 × 10^5^ PFU of H9N2 virus and then intranasally inoculated with 1 × 10^6^ CFU of S. pneumoniae at 7, 14, 21, 28, 35, or 42 dpi. The mice were sacrificed at 6 h after pneumococcal infection, and whole lung tissues were harvested to analyze the bacterial load as described below.

### Plaque assay.

At the indicated time points after H9N2 virus inoculation, mice were sacrificed by cervical dislocation, and the whole lungs were collected aseptically in sterile tubes and homogenized in 1 ml of sterile saline. The lung homogenates were centrifuged, and then the supernatants were filtered using a 0.22-μm filter membrane. Then, H9N2 virus concentrations in lung tissues were determined by plaque assay as described previously (59). Briefly, adsorption of 0.5 ml of 10-fold serial dilutions of viral samples was performed on Madin-Darby canine kidney monolayers, which were overlaid with a 1% final concentration of agarose and a 1 μg/ml final concentration of TPCK (tosylsulfonyl phenylalanyl chloromethyl ketone) trypsin. After 72 h, cells were fixed with 4% formaldehyde and stained with 2% crystal violet to detect plaques. Viral titer (in PFU per milliliter) was calculated as plaque counts/(0.5 ml × dilution factor of the sample).

### Histopathological examination of lung tissues.

At the indicated time points after H9N2 virus or S. pneumoniae inoculation, the left lobes of the lungs were removed immediately after euthanasia, fixed in 4% paraformaldehyde, and then embedded in paraffin. Fixed sections (3 to 5 μm) of paraffin-embedded lungs were stained with hematoxylin-eosin (H&E) for examining histopathological alterations in the lung parenchyma under a light microscope. Based on the extent of histopathological alterations, including peribronchial inflammation, intra-alveolar inflammation, perivascular inflammation, bronchial epithelial shedding, and intra-alveolar fibrin exudation, three sections per lung were blindly scored on a scale of 0 (no lung area affected) to 4 (100% of the lung area affected) by an experienced pathologist, as described previously (60, 61). Results were expressed as percentage of lung area affected, calculated as (total scores of three sections/3) × 25%.

### Measurement of bacterial loads in lung tissues.

At the indicated time points after S. pneumoniae inoculation, mice were sacrificed by cervical dislocation, and the whole lungs were collected aseptically in sterile tubes and homogenized in 1 ml of sterile PBS. Then the volume of the lung homogenate was increased to 3 ml with sterile PBS. Finally, bacterial loads in lung tissues were determined by plating 0.1 ml of 10-fold serial dilutions on blood agar plates, and colonies were counted after incubating for 24 h at 37°C. Bacterial loads (CFU per lung) were calculated as (colony counts × 3 ml)/(0.1 ml × dilution factor of the sample) (58).

### Measurement of cytokine levels in lung tissues.

Lung homogenates were centrifuged and the supernatants were collected and stored at −80°C until measurement of cytokine levels. The levels of keratinocyte chemoattractant (KC), mouse macrophage inflammatory protein-2 (MIP-2), and interleukin-1β (IL-1β) were measured using mouse Quantikine enzyme-linked immunosorbent assay (ELISA) kits (R&D Systems, USA). The levels of interleukin-6 (IL-6), tumor necrosis factor alpha (TNF-α), and interleukin-10 (IL-10) were measured using mouse Quantikine ELISA kits (Solarbio, China). All of the above-mentioned cytokines were measured according to the manufacturer’s instructions.

### Statistical analysis.

All data are presented as means and standard errors of the means (SEM). Data between two groups were analyzed by using two-tailed unpaired Student’s *t* test. Data among multiple groups were analyzed by using ordinary one-way analysis of variance (ANOVA) followed by Tukey’s multiple-comparison test. Survival data were analyzed by using a log-rank (Mantel-Cox) test. All statistical analyses were performed using GraphPad Prism 8 software (GraphPad Software, USA). Results with *P* values of <0.05 were considered significant.
